# Plant-Growth-Promoting Rhizobacteria Modulate Carbohydrate Metabolism in Connection with Host Plant Defense Mechanism

**DOI:** 10.3390/ijms25031465

**Published:** 2024-01-25

**Authors:** Fan Su, Bin Zhao, Sandrine Dhondt-Cordelier, Nathalie Vaillant-Gaveau

**Affiliations:** 1 Institute of Agro-Product Safety and Nutrition, Tianjin Academy of Agricultural Sciences, Tianjin 300071, China; fansu.agroscience@hotmail.com; 2 State Key Laboratory of North China Crop Improvement and Regulation, College of Plant Protection, Hebei Agricultural University, Baoding 071001, China; bdzhaobin@126.com; 3 Unité de Recherche Résistance Induite et Bioprotection des Plantes—USC INRAE 1488, Université de Reims Champagne Ardenne, 51100 Reims, France; sandrine.cordelier@univ-reims.fr

**Keywords:** plant-growth-promoting rhizobacteria, photosynthesis, carbohydrate metabolism, defense mechanism

## Abstract

Plant-growth-promoting rhizobacteria (PGPR) could potentially enhance photosynthesis and benefit plant growth by improving soil nutrient uptake and affecting plant hormone balance. Several recent studies have unveiled a correlation between alterations in photosynthesis and host plant resistance levels. Photosynthesis provides materials and energy for plant growth and immune defense and affects defense-related signaling pathways. Photosynthetic organelles, which could be strengthened by PGPR inoculation, are key centers for defense signal biosynthesis and transmission. Although endophytic PGPRs metabolize plant photosynthates, they can increase soluble sugar levels and alternate sugar type and distribution. Soluble sugars clearly support plant growth and can act as secondary messengers under stressed conditions. Overall, carbohydrate metabolism modifications induced by PGPR may also play a key role in improving plant resistance. We provide a concise overview of current knowledge regarding PGPR-induced modulation in carbohydrate metabolism under both pathogen-infected and pathogen-free conditions. We highlight PGPR application as a cost-saving strategy amidst unpredictable pathogen pressures.

## 1. Introduction

Plants, as autotrophs, produce their own chemical energy and organic compounds from sunlight, carbon dioxide and water through photosynthesis. Photosynthesis is a series of photochemical (light-dependent reactions, electron transport from water to oxidized form of nicotinamide adenine dinucleotide phosphate, NADP^+^) and biochemical (independent of light, CO_2_ fixation) reactions. The two reaction groups occur in different locations in chloroplasts, thylakoid membranes, and stroma, respectively. Light energy absorbed by pigments initiates primary photochemical reactions in the two interconnected photosystems (PSs) PSI and PSII, which involve several redox components. This is followed by reactions resulting in the production of stable organic compounds (carbohydrates) from CO_2_. Photosynthetic efficiency is positively correlated with crop yield, and not yet optimized for industrial agriculture [1].

**Figure 1:**
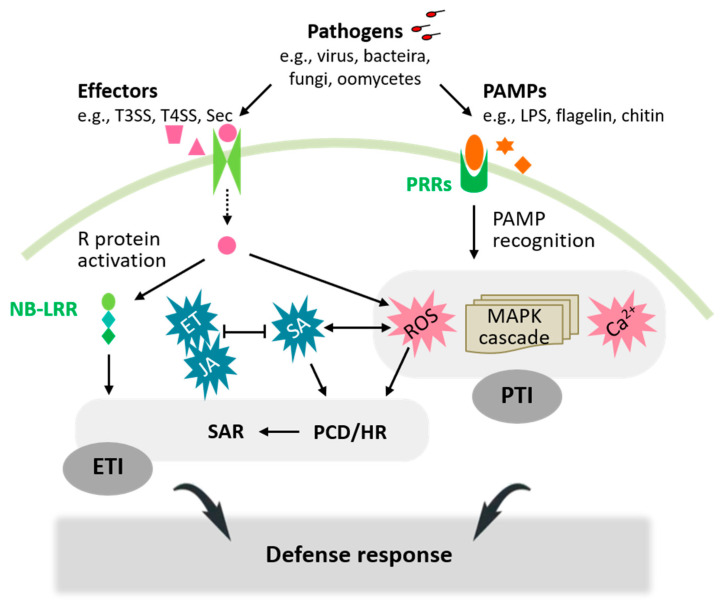
Schematic representation of plant defense activation in response to pathogen infection. The plant immune response is a sophisticated defense mechanism involving two primary layers: PTI and ETI. Pattern-triggered immunity inhibits the growth of most pathogens through the recognition of PAMPs by PRRs, activating signaling pathways such as Ca^2+^ and ROS signaling, as well as the MAPK cascade. However, certain pathogens can release pathogenic effectors to interfere with PTI, resulting in effector-triggered susceptibility. Effector-triggered immunity is initiated by the interaction of R proteins, possessing a conserved NB-LRR domain, with specific pathogenic effectors. This recognition often leads to the activation of PCD/HR at the infection site, effectively repressing the growth of pathogens once again. Both ETI and PTI engage the defense-related hormone signaling pathways, primarily SA and JA/ET, inducing downstream transcription factors. The interaction of SA- and JA/ET-dependent signaling pathways is generally considered to be antagonistically involved in the regulation of immune response to pathogens with different lifestyles. The signaling pathway involving SA is generally responsible for avoiding biotrophs or hemibiotrophs, while JA/ET-mediated defense responses are more effective against necrotrophic pathogens. Full arrows depict positive regulation, open blocks negative regulation, and broken arrows transport. Abbreviations: Ca^2+^—calcium; ET—ethylene; ETI—effector-triggered immunity; HR—hypersensitive response; JA—jasmonic acid; LPS—lipopolysaccharide; MAPK—mitogen-activated protein kinase; NB-LRR—nucleotide-binding leucine-rich repeat; PAMP, pathogen-associated molecular pattern; PCD—programmed cell death; PRRs—pattern recognition receptors; PTI—PAMP—triggered immunity; R—resistance; ROS—reactive oxygen species; SA—salicylic acid; SAR—systemic acquired resistance; Sec—general protein secretory; T3SS—type III secretion system; T4SS—type IV secretion system.

In nature, plants are constantly exposed to pathogens. Plant pathogens are generally classified as biotrophs, hemibiotrophs or necrotrophs depending on their lifestyles and infection strategies. Biotrophic/hemibiotrophic pathogens require living tissue for their growth and reproduction. Nevertheless, hemibiotrophs induce plant tissue damage in the later stages of infection [2], while necrotrophs kill host tissue early in infection and feed on the dead tissues. After pathogen infection, the coordination of the plant defense response is intricately regulated to confer resistance against a variety of pathogens (Figure 1). In leaf necrotrophic pathogens, it is easy to imagine that the host plant’s high-protein photosynthetic apparatus is destroyed during their propagation [3,4]. However, photosynthetic pigment degradation, photosynthetic electron transport disruption and/or alteration of downstream metabolic reactions occur not only during leaf necrotrophic pathogen infestations but also in biotrophic/hemibiotrophic pathogen infections [4,5,6]. These disruptions extend to situations where pathogens infect plant roots [6,7]. Crucially, photosynthesis metabolism and carbohydrate distribution are not only affected by pathogens, but also shaped by plant-stress-adaptive mechanisms or defense mechanisms [5,8,9].

Plant-growth-promoting rhizobacteria (PGPR) naturally live in the closely adhering soil interface (rhizosphere) and can gradually colonize root surfaces (rhizoplane) as epiphytes, or even internal root tissues (endophytes). These bacteria can directly promote plant growth by improving nutrient acquisition through nitrogen fixation, siderophore production or soil nutrient solubilization [10,11,12], and/or modulating plant hormone balance [13,14,15]. These effects on nutrient uptake, plant hormone levels, and their signaling pathways are likely to alter photosynthesis and carbon flux. Increasingly, more PGPR strains have been shown to potentially increase photosynthetic pigment and soluble sugar contents, enhance photosynthesis, and modify sugar distribution. Increased photosynthetic capacity and PGPR efficiency could help offset stress adaptation and defense costs during pathogen infection. However, there is limited understanding of the contribution of PGPR-mediated carbohydrate metabolism to plant resistance against phytopathogens. Thus, we provide a review of current knowledge of PGPR-induced modulations in photosynthesis and carbohydrate distribution, highlighting their connection with host plant defense mechanisms and overall plant productivity.

## 2. PGPR Inoculation Influences Carbohydrate Metabolism

Evidence for increased yield in response to high levels and activity of photosynthesis-related proteins [16,17] or CO_2_ enrichment [18,19] has been shown consistently, providing compelling evidence that yields can be increased through photosynthetic improvement. Comprehensive studies have been conducted on the interactions between PGPR and plants at transcriptomic, proteomic, and metabolomic levels. The influence of PGPR on gene transcription and protein activity associated with photosynthesis has been demonstrated [15,20,21,22]. Furthermore, PGPR affects aspects of carbohydrate metabolism, such as pigment content, photosystem efficiency, CO_2_ fixation, and carbohydrate level (Table 1).

### 2.1. Photosynthetic Pigments

Photosynthetic pigments, including chlorophyll (Chl) a, Chl b, and carotenoids, play pivotal roles in both light harvesting and protection of the photosynthetic system from excessive light damage. Their levels are an important plant physiological state indicator and can be used to estimate photosynthetic activity. Plant Chl levels can be boosted by inoculation with nitrogen-fixing, phosphate-solubilizing, or siderophores-producing PGPR [11,12,13,23,24]. Comparison of grapevine rootstocks bacterized with different PGPR strains (*Azospirillum brasilense* Sp245, *Burkholderia gladii* BA-7, *Bacillus subtilis* OSU-142 or *B. megatorium* M-3) revealed that PGPR-induced Chl accumulation was significantly correlated with bacterial ability to stimulate host plant nutrient acquisition (e.g., N, P, K, Ca, or Mg) [10]. A recent meta-analysis assessing the benefits of various microbial inoculants on crop yield confirms that PGPR enhance crop productivity by improving essential nutrients uptake (N, P, K) and hence increasing chlorophyll levels [24].

**Figure 2:**
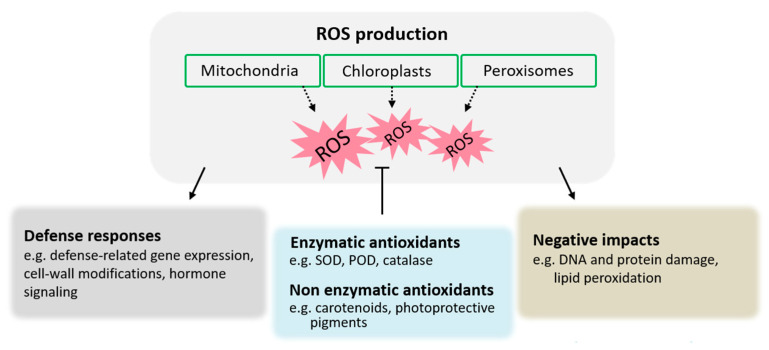
Activation and effects of ROS production. Pathogens release elicitors recognized by PRRs, activating pattern-triggered immunity. Concurrently, effectors induce effector-triggered immunity through recognition by resistance proteins. This recognition prompts ROS production in chloroplasts, mitochondria, and peroxisomes. A low ROS level enhances defense mechanisms, including changes in gene expression, cell wall reinforcement, and cell death initiation, collectively influencing the plant’s immune status and physiology. Despite potentially damaging effects on proteins and DNA, plants activate antioxidant agents to regulate ROS and bolster their defense systems. Full arrows depict positive regulation, open blocks negative regulation, and broken arrows transport. Abbreviations: POD—peroxidase; PRRs—pattern recognition receptors; ROS—reactive oxygen species; SOD—superoxide dismutase.

PGPR strains secreting 1-aminocyclopropane-1-carboxylate (ACC) deaminase can hydrolyze the ethylene precursor ACC, thereby reducing the ethylene amount [25]. Ethylene serves as a pivotal plant growth regulator involved in several physiological processes (germination, root initiation, leaf and flower senescence, fruit ripening, and organ abscission) and stress signaling [26]. Low ethylene levels benefit plant growth and development, while excess ethylene is synthesized in response to an environmental stress stimulus, pathogen, or insect [26,27]. Inoculation with ACC deaminase-producing PGPR results in increased Chl contents, biomass, and crop yield [28,29]. *Pseudomonas fluorescens* YsS6 and *P. migulae* 8R6 are the ACC deaminase-producing strains, and their ACC-deaminase-deficient mutants lose the ability to increase Chl content and biomass in tomato plants [28]. Seed bacterization by *A. brasilense* Sp245 increases not only Chl content but also carotenoid and photoprotective pigment (violaxanthine, antheroxanthine, and zeaxanthin in wheat and anthocyanin in Arabidopsis) contents [14,30]. Photoprotective pigments, such as anthocyanins, do not engage in light capture, but they reduce photoinhibition and photodamage by shielding against visible radiation and/or eliminating reactive oxygen species (ROS) through their antioxidant properties (Figure 2) [31]. Using the Arabidopsis mutant *aba2-1*, which is defective in the synthesis of abscisic acid (ABA), Cohen et al. [14] suggested that an increase in pigment contents may be due to the accumulation of endogenous ABA induced by *A. brasilense* Sp245. Exogenous ABA treatment can augment photosynthetic and photoprotective pigments in grapevine [32], wheat [33], and maize [34].

**Table 1:** PGPR-induced modifications in carbohydrate metabolism.

PGP Traits	PGPR Involved	Host Plant	Effect	Ref
Nitrogen fixation	*Azospirillum* sp. Sp7 *Bacillus sphaericus* UPMB10	Banana plantlets	Chl accumulation	[11]
	*Aeromonas hydrophila* P73 *Serratia proteamaculans* 1–102 *S. liquefaciens* 2–68 *Pseudomonas putida* G11-32	Soybean	Pn enhancement	[35]
Siderophore	*Pseudomonas* sp. RRLJ 008	Eggplant, cabbage, tomato, bean, and kohlrabi	Chl accumulation	[12]
Siderophore and phosphorous solubilization	*B. pumilus* S4	Runner bean	Chl accumulation, Pn enhancement, boosted POD and SOD activity during periods of intense photosynthesis	[13]
ACC deaminase	*P*. *fluorescens* YsS6 *P. migulae* 8R6	Tomato	Chl accumulation	[28]
IAA	*B. mycoides* S7	Runner bean	Chl accumulation, Pn enhancement, boosted POD and SOD activity during periods of intense photosynthesis	[13]
	*Paraburkholderia phytofirmans* PsJN	Grapevine plantlets	Soluble sugar and starch accumulation	[36,37]
		Maize	Chl accumulation, PSII efficiency enhancement	[23]
ABA	*A. brasilense* Sp245	Wheat, Grapevine Arabidopsis	Chl, arotenoid, and photoprotective pigment accumulation	[10,14,30]
Volatile compounds	*B. subtilis* GB03	Arabidopsis	Chl accumulation, PSII efficiency enhancement, endogenous glucose modulation, overexpression of genes involved in photosynthesis	[15]
	*B. amyloliquefaciens FZB42*	Arabidopsis	Regulation of genes related to photosynthetic light harvesting	[21]
	*B. subtilis* JS	Tobacco	Regulation of genes involved in photosynthesis	[22]

Abbreviations: ABA—abscisic acid; ACC—1-aminocyclopropane-1-carboxylate; Chl—chlorophyll; IAA—indoleacetic acid; PGP—plant-growth-promoting; PGPR—plant-growth-promoting rhizobacteria; Pn—net carbon assimilation; POD—peroxidase; PSII—photosystem; SOD—superoxide dismutase.

### 2.2. Photosystem Efficiencies

Higher photosynthetic pigment content favors better light absorption at the beginning of the photosynthesis mechanism, normally resulting in increased photosystem activity. One of the most measured parameters for characterizing photosynthetic capacity is PSII activity, including electron transport rate (ETR), PSII maximal yield (Fv/Fm), and PSII effective quantum yield [38]. These different PSII activity parameters are obtained by measuring chlorophyll fluorescence. Chlorophyll fluorescence is a powerful, rapid, and minimally invasive indicator of plant health, providing specific signatures for the diagnosis of distinct diseases [38] and environmental stress [39]. By monitoring in vivo chlorophyll fluorescence, enhanced PSII activity was detected in plants colonized by PGPR strains such as *A. brasilense* Sp245 [14,40], *B. subtilis* GB03 [15], or *Paraburkholderia phytofirmans* PsJN [23] with the potential to increase photosynthetic pigment levels. However, no significant PSII or PSI modification efficiency was observed in *P. phytofirmans* PsJN-colonized *A. thaliana* [41] or grapevine plantlets [36] following Chl level variation. Rincón’s studies further confirmed that plant host species significantly influence PGPR effects on photosynthetic efficiency. Inoculation with *P. fluorescens* Aur6 can promote PSII activity (ETR and Fv/Fm) in drought-resistant plants (*Pinus halepensis* Mill.) but not in drought-sensitive plants (*Quercus coccifera* L.) [42]. The fluorescence parameter Fv/Fm, which reflects the maximum quantum efficiency of PSII photochemistry, generally decreased under drought stress, but maintained a relatively high level in drought-resistant varieties [43,44].

Beyond photosynthetic or photoprotective pigment levels, photosystem stability also relies on various auxiliary proteins (e.g., light-harvesting complex proteins) and a series of enzymatic reactions (e.g., phosphorylation and dephosphorylation of PSII core and antenna proteins) [45]. Arabidopsis genes encoding photosynthesis–antenna proteins or related to photosynthetic light harvesting were up-regulated by volatile compounds released by *B. amyloliquefaciens* FZB42 at the mature stage (first bud appearance) but down-regulated at seedling stage (10 rosette leaves) [21]. Arabidopsis seedlings co-cultured with *B. subtilis* GB03, but without any contact, displayed accumulated Chl content and chloroplast number, enhanced Fv/Fm, and an overexpression of genes involved in photosynthesis [15]. These effects are achieved by endogenous glucose and ABA level modulations with the help of *B. subtilis* GB03 volatiles. In Arabidopsis mutants deficient in glucose signaling (*gin2* and *gin1/ABA2*) or grown in an ABA-enriched medium, *B. subtilis* GB03 volatiles cannot stimulate Chl content accumulation or photosystem efficiency [15]. Therefore, the magnitude of PGPR-induced modification on photosystem efficiencies is probably related to host plant physiological characteristics.

### 2.3. CO_2_ Fixation

Promoting photosynthesis can be facilitated by PGPR through the modulation of gas exchange, a process occurring between plants and the atmosphere in the leaf through stomata. During photosynthesis, plants consume CO_2_ and release oxygen, while the reverse happens during plant respiration. Rhizobia symbiont activity in the nodules of cultivated peas is directly related to carbon nutrition [46]. In Fabaceae and cereals, *Bradyrhizobium* strains stimulate root respiration by increasing stomatal conductance and transpiration [47]. Stomatal conductance is a numerical measure of the speed of passage of either water vapor or CO_2_ through the stomata, and a high stomatal conductance indicates that the stomata are open. Observations show a significant relationship between stomatal conductance and photosynthetic capacity [48,49]. Rhizobium population growth requires more CO_2_ [50], which may account for the increased availability of lumichrome-induced CO_2_ [51]. However, the modulation mechanism of carbon fluxes by lumichrome is still unknown [52].

Nitrogen-fixing PGPR strains (e.g., *Serratia proteamaculans*, *S. liquefaciens*, and *P. putida*) favored soybean leaf net carbon assimilation (Pn) at different growth stages, even before nitrogen fixation onset [35]. However, the inoculation of the PGPR strain secreting auxin (indole-3-acetic acid, IAA, *B. mycoides*) or producing siderophores and solubilizing phosphate (*B. pumilus*) triggered an increase in Pn and plant transpiration with a negative effect on nitrogen fixation [53]. These findings suggest that nitrogen fixation is not always essential to promote photosynthesis; rather, the main mechanism for improving the photosynthesis of these tested rhizobacteria is based on the physiological modification of the plant. For example, PGPR presence increases antioxidant enzyme activity (superoxide dismutase, SOD, and peroxidase, POD) during intense photosynthesis periods [13,53]. These enzymes protect chloroplasts from oxidative stress by scavenging ROS [54].

### 2.4. Plant Host Carbohydrate Levels

The genomes of 1160 plant-associated bacteria, isolated from *A. thaliana*, barley, maize, wheat, cucumber, and poplar, were compared with those of 2677 bacteria isolated from non-plant environments that shared a common ancestor [55]. This large-scale comparative genomics study revealed that genomes of plant-associated bacteria possess more genes involved in carbohydrate sensing, metabolism, or transport than those from non-plant-associated bacteria [55]. For *B. cereus* PGPR strains isolated from alfalfa, wheat, haw, and forest soil, the function of their strain-specific genes is more related to carbohydrate metabolism and transcription than plant-growth-promoting traits [56]. These studies reveal a significant diversity in bacterial communities among various plant species and even within different cultivars of the same species. The findings also highlight that the evolution of bacterial carbohydrate metabolism is a main factor in their adaptation to plant associated life.

Photosynthetic yields are strongly correlated with chloroplast function, stomatal conductance, and net CO_2_ assimilation rate. Treatment with *B. subtilis* JS volatile compounds up-regulated tobacco genes related to photosynthesis pathway, like those encoding Chl a/b binding protein, chloroplast SBPase, and the photosynthate transport related gene [22]. Root inoculation with PGPR can lead to soluble sugar (e.g., fructose, glucose, maltose, trehalose) accumulation in leaves and/or roots [36,37,57]. Inoculation of grapevine plantlets by the IAA producer *P. phytofirmans* PsJN increased not only leaf total soluble sugars concentration but also starch level [36]. Auxin is known to be required for starch synthesis in higher plants [58,59]. An evident correlation between *A. brasilense* IAA production and starch accumulation in its host microalgae *Chlorella sorokiniana* has been proven [60]. Thereby, the theoretical consideration is that carbohydrate metabolism can be modulated by the PGPR-secreted hormone.

## 3. PGPR-Induced Alterations in Photosynthesis and Carbon Fluxes Contribute to Plant Defense

Plant protection against pathogens by PGPR can occur either through an antagonistic interaction or by activating defense mechanisms that lead to induced systemic resistance (ISR). Strains with ISR-activating potential are primarily identified in the genera *Bacillus*, *Pseudomonas*, and *Serratia*, which are extensively studied and increasingly marketed. However, PGPR inoculation may not result in consistent changes in the systemic immune response in the absence of pathogens [61,62,63]. Upon being challenged by an attacker, plants receive PGPR warning signals and thus could display an enhanced perception of the attacker and mount a faster and stronger immune response [62,64]. In addition to priming plants’ defense responses against pathogens, certain PGPR strains are helpful for maintaining photosynthesis and even enhancing its capacity and efficiency [65,66,67]. Mitigating damage to photosynthesis and the energy loss caused by pathogen invasion or plant defense ensures plant growth in the face of stress.

### 3.1. Maintaining Chloroplast Structure and Function

Pepper leaf infection by the necrotrophic fungus *Alternaria alternata* significantly reduced photosynthetic pigments and chloroplasts, collapsed chloroplasts, and changed photosynthetic chemistry [67]. Significant photosynthetic pigment loss commonly results from pathogen infection and manifests as chlorosis [68,69,70]. Photosynthesis and photosynthate production may be restricted as a consequence of low levels of photosynthetic pigments. During common bean plant infection by the hemibiotrophic pathogen *Colletotrichum lindemuthianum*, the photosynthetic rate was linearly correlated with total Chl and carotenoid contents [68]. Soliman et al. [67] employed *B. amyloliquefaciens* RaSh1 as a biocontrol agent against *A. alternata* on pepper plants. *B. amyloliquefaciens* RaSh1 root inoculation increased Chl a and b and carotenoids levels and promoted plant growth, regardless of whether or not the plants were infected with *A. alternata*. Moreover, Chl a and b amounts were significantly and positively correlated with several plant growth parameters, including dry weight and root or shoot length. Further investigations into the impact of plant–PGPR interactions on disease resistance affirmed that PGPR strains can enhance Chl contents in both disease-free and diseased plants while fostering plant growth [7,65,66,71].

**Figure 3:**
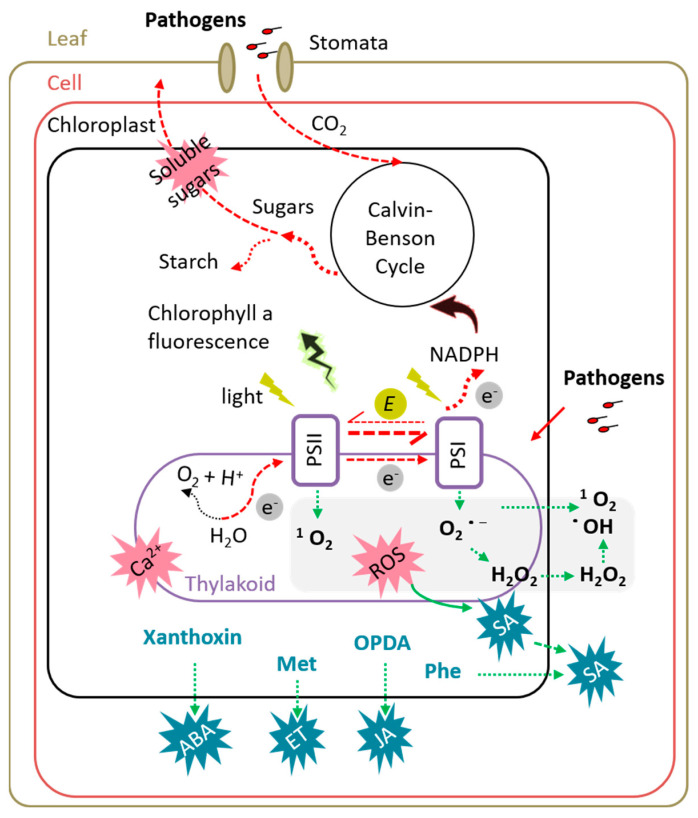
Role of photosynthesis in plant defense: sensor, reactor, and energy source. Pathogens primarily invade leaves through natural openings, such as stomata. When stomatal cells detect microbial-associated molecular patterns, stomata close to prevent further pathogen penetration. Moreover, the perception of live microbes by plant cells can induce a rapid production or transmission of defense signals in chloroplasts, including Ca^2+^, ROS, and hormones (ABA, ET, JA, and SA). Transmission of these immune signals leads to defense-related gene expressions and the establishment of local and systemic immunity. However, stomatal closure as an early defense response limits not only pathogen invasion but also CO_2_ input, resulting in photosynthetic activity restriction. Meanwhile, pathogen infection disrupts chloroplast ultrastructure and function, impacting photosynthetic chemistry. The negative impact of pathogens on net CO_2_ assimilation rate, stomatal conductance, and chloroplast function results in a decreased photosynthetic yield. Full arrows depict interactions, broken arrows transport, and dotted arrows metabolic reactions. Additionally, red arrows indicate the negative impact or reduction resulting from pathogen invasion, while green arrows represent positive effects or activation. The biosynthesis of the lipid-derived hormone JA begins in the chloroplast but is completed in the peroxisome. Abbreviations: ABA—abscisic acid; Ca^2+^—calcium; E—photon; e^−^—electron; ET—ethylene; JA—jasmonic acid; Met—methionine; NADPH—nicotinamide adenine dinucleotide phosphate; OPDA—12-oxo-phytodienoic acid; Phe—phenylalanine; PSI—photosystem I; PSII—photosystem II; ROS—reactive oxygen species; SA—salicylic acid.

Using transmission electron microscopy, irregular shapes and chloroplast leakage have been widely detected in plants under pathogenic stress [67,72,73,74]. Chloroplasts are integral to photosynthesis and also crucial in regulating plant immune responses by synthesizing and/or transmitting defense signals, including Ca^2+^, ROS, and phytohormones like ethylene, jasmonic acid (JA), salicylic acid (SA), and ABA [75,76]. Pathogens can affect plant defense signaling by manipulating chloroplast structure and function (Figure 3) [77,78,79]. The *P. syringae* virulence effector HopI1 binds to plant 70 kDa heatshock proteins (Hsp70) through its C-terminal J domain, stimulating Hsp70 ATP hydrolysis activity and forming large complexes with cytosolic Hsp70 at chloroplasts [80]. Chloroplast-localized HopL1 disrupts thylakoid structure and hinders SA accumulation, ultimately enhancing bacterial virulence by undermining plant defenses [78]. Chloroplast ultrastructure is preserved with PGPR under abiotic stress, such as alkaline conditions [81], and high [82] or freezing temperature [41]. Furthermore, *B. amyloliquefaciens* RaSh1, *P. fluorescens* 89B61, and *S. marcescens* 90-166 preserved chloroplast ultrastructure and function during pathogen infection processes in pepper and cucumber, respectively [67,83]. Avoiding chloroplast damage during stress conditions could be a strategy to improve plant resistance as well as promote plant growth [77,84].

### 3.2. Maintaining the Balance between ROS Production and Antioxidant Defense

Reactive oxygen species, such as hydrogen peroxide (H_2_O_2_), singlet oxygen (^1^O_2_), superoxide anion radicals (O_2_^˙−^), and hydroxyl radicals (˙OH), act as common messengers in plant responses to developmental processes and biotic and abiotic stresses [85]. Within the chloroplast, oxygen, as the source of all ROS, is consistently produced and eliminated by reduction or assimilation. When light absorption exceeds photosynthetic electron transport capacity, ^1^O_2_ can be generated at PSII, while O_2_^˙−^ is usually produced at PSI and results in photoinhibition [86]. Both ^1^O_2_ and O_2_^˙−^ are unstable but greatly impact photosynthesis [76,86]. Moreover, O_2_^˙−^ can be reduced to H_2_O_2_ via plastoquinol or dis-mutated to H_2_O_2_ either spontaneously or by SOD [85,87]. Upon microbial invasion, the recognition of microbe/pathogen-associated molecular patterns activates a plant signaling cascade through resistance genes. This cascade induces ROS generation via mitochondrial and chloroplastic electron transport chains, as well as peroxisomal photorespiration. Low-level ROS production contributes to the activation of plant defense mechanisms against a diverse range of pathogens, such as hypersensitive response, cell wall reinforcement, and SA-dependent defense pathways [85].

For a successful infection, pathogens may remodel thylakoid membranes, disrupt electron transport, and minimize chloroplastic ROS production. Arabidopsis leaf infection by *P. syringae* pv. *tomato* DC3000 suppresses Fv/Fm and transiently increases photochemical quenching of PSII (qL) and non-photochemical quenching (NPQ), but the disarmed *P. syringae* pv. *tomato* DC3000 *hrpA* mutant does not [77]. High qL values reveal an increased fraction of open PSII centers and high oxidation state of the primary PSII quinone receptor [38]. This shift implies slowed electron transfer from PSII, hinting at compromised PSII function. High NPQ values indicate a proactive protective response to stress, involving the dissipation of excess excitation energy as heat [38]. Therefore, de Torres Zabala et al. [77] suggested that the hemibiotrophic bacterium *P. syringae* pv. *tomato* DC3000 manipulates PSII to prevent a chloroplastic ROS burst and its downstream defense responses. The flagellin-derived peptide fragment Flg22 plays a pivotal role in eliciting plant defense responses. It serves as a crucial tool for studying molecular pattern-triggered immunity and exploring plant responses to bacteria. Upon exposure to Flg22 derived from PGPR *P. phytofirmans* PsJN, grapevine cells exhibit immune responses, including weak H_2_O_2_ accumulation, transient SA production, overexpression of some defense genes, and extracellular alkalinization [88,89]. However, grapevine plantlet bacterization with *P. phytofirmans* PsJN does not activate H_2_O_2_ production in leaves until *Botrytis cinerea* infection [63], highlighting that PGPR can prime systemic production of ROS vis-à-vis a foliar pathogen.

Under stressed conditions, when ROS overproduction exceeds existing antioxidative defense mechanisms, oxidative stress may occur. High photoprotective pigment levels induced by PGPR enhance host plant antioxidant ability and help to avoid oxidative damage [14,32]. Moreover, tomato root inoculation with *B. subtilis* PS1-3 or *P. fluorescens* PS2-6 promotes plant growth and yield with an improved photosynthetic performance and increases antioxidant enzyme activity (e.g., SOD, POD, and catalase) in leaves [90]. When *F. oxysporum* leaf infection occurred, these defense enzyme activities were more strongly boosted in plants associated with *B. subtilis * PS1-3 or *P. fluorescens* PS2-6 than in controls [90]. Superoxide dismutase converts O_2_^˙−^ into H_2_O_2_ and then catalase and POD convert two peroxide molecules into two water molecules and a dioxygen molecule [76]. A meta-analysis of 561 studies revealed increased SOD and catalase activities in plants inoculated with PGPR under salt stress [91]. Supporting this, Ali et al. [92,93] recently reported that the PGPR strains *B. mycoides* PM35 and *Enterobacter cloacae* PM23 improve maize plant growth and survival under salinity stress, reducing ROS production and decreasing membrane injury through the production of various antioxidants, such as ascorbic acid and the redox enzymes ascorbate peroxidase, POD, and SOD. These results imply that PGPR colonization could potentially maintain the balance between ROS production and antioxidant defense in plants.

### 3.3. Redistribution of Sugars

**Figure 4:**
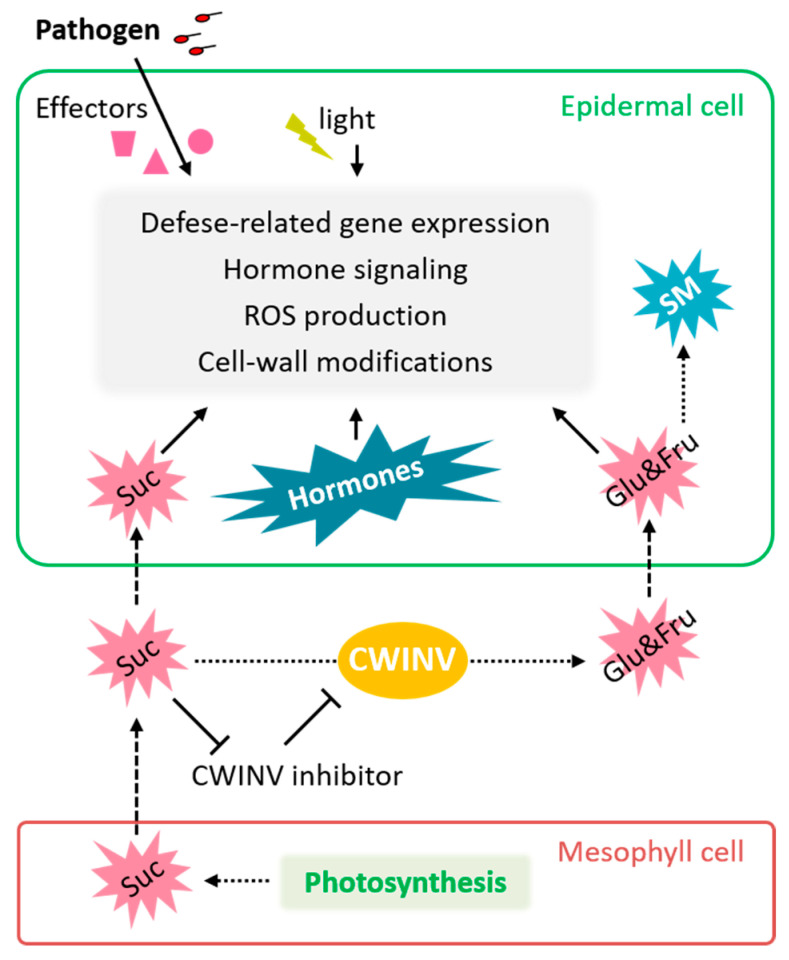
Function and regulation of CWINV and soluble sugars in plant defense. Sucrose produced through photosynthesis is exported into the apoplast, where CWINV hydrolyzes it into fructose and glucose. These hexoses are then metabolized to support respiration or the synthesis of secondary metabolites like callose or phenolic compounds. Pathogen infection boosts CWINV expression or activity by down-regulating proteinaceous CWINV inhibitors, leading to increased sucrose cleavage. This results in a continuous efflux of sucrose from the phloem and the establishment of an additional sink. Furthermore, fructose, glucose, and sucrose act as signaling molecules in plant defense, responding to factors like hormones, light, and circadian regulation. Full arrows depict interactions, broken arrows transport, and dotted arrows metabolic reactions. Abbreviations: CWINV—cell wall invertase; Frc—fructose; Glc—glucose; ROS—reactive oxygen species; SM—secondary metabolites; Suc—sucrose.

The primary photosynthate sucrose is transported from photosynthesizing leaves (sources) to non-photosynthetic plant tissues (sinks) via the phloem to provide growth substrate [94]. Sucrose exported into the apoplast is hydrolyzed by cell wall invertase (CWINV) to fructose and glucose to maintain growth at specific sites (Figure 4). By untargeted metabolomic analysis, van de Mortel et al. [95] found that 50 metabolites, including glucose and fructose, were differentially regulated in plants treated with ISR-eliciting PGPR *P. fluorescens*. Increased soluble sugar levels support plant growth and can also serve as secondary messengers to induce defense response [96,97]. During plant–pathogen interactions, infected leaf sites not only fail to sustain photosynthate export but also increase carbohydrate demand [4,96]. The redistribution of sugars caused by pathogen infection is related to several enzymes, such as CWINV (Figure 4). The activity of CWINV is potentially increased by pathogen attacks, which leads to a continued efflux of sucrose from the phloem and results in an additional sink [4,8,96]. Tomato root inoculation with the ISR-eliciting PGPR *P. pseudoalcaligenes* did not affect CWINV activity [98], while infection with the fungal pathogen *Sclerotium rolfsii* caused a strong increase in CWINV activity in tomato plants with and without *P. pseudoalcaligene*, although it was a bit lower in the former [98]. After a challenge with *S. rolfsii*, plants associated with *P. pseudoalcaligene* showed an increased leaf fructose amount and slightly elevated mRNA levels of the defense-related genes *PR2* and *PR3* [98]. Fructose plays a role in Arabidopsis’s response to *B. cinerea* through specific pathways associated with ABA and ethylene signaling [97].

### 3.4. Fine-Tuning Trade-Offs between Defense and Carbohydrate Metabolism

Exogenous application of a defense-related hormone, such as SA or JA, improves plant disease resistance but negatively affects photosynthesis, plant growth, and development [99,100]. Millimolar SA treatment decreases Chl contents, hinders photosynthetic protein ribulose-1,5-bisphosphate carboxylase/oxygenase (RuBisCO) synthesis, and inhibits carbonic anhydrase activity and the net CO_2_ assimilation rate [100,101]. Meanwhile, Arabidopsis mutants that express defense responses have impeded growth and reduced fertility, while mutants with deficient defense signaling pathways grow taller [102,103]. With limited resources, balancing the costs and benefits of defense mechanisms is critical to optimizing plant fitness and survival under stress. By quantitative analysis of ^15^N–labeled proteins, Ullmann-Zeunert et al. [104] found that RuBisCO and total soluble protein decreased with increased defense metabolites (nicotine and phenolamide) in tobacco shoots under persistent herbivory infection. However, ^15^N flux tracking results indicate that nitrogen used for phenolamide synthesis does not originate from RuBisCO but arises from nitrogen recently assimilated by the plant [104]. Ullmann-Zeunert’s team confirmed this finding using a RuBisCO-silenced transgenic tobacco line, as*RUB* [105]. Compared with the wild type, phenolamide biosynthesis was not restricted in the as*RUB* plants after herbivory [105]. Thus, the photosynthetic protein reduction upon pathogen challenge may be related to a “survival strategy” rather than the result of resource constraints caused by defense activation.

Strains, which have the potential to promote photosynthesis, may enable plants to improve resistance while reducing the magnitude of fitness costs, even to a negligible level [20,65,67,106]. *Paenibacillus polymyxa* E681 inoculation significantly improves Arabidopsis disease resistance against *B. cinerea* with improved photosynthesis and plant growth [20]. Proteomics showed that Arabidopsis root inoculation with *P. polymyxa* E681 up-regulated the accumulation of 36 proteins involved in photosynthesis, amino acid metabolism, antioxidants, hormone signaling, and defense and stress responses [20]. Similarly, various PGPR *Bacillus* strains, such as *B. subtilis* BERA71 [71] and *B. aryabhattai* SRB02 [65], as well as *B. amyloliquefaciens* RaSh1 [67] and RWL-1 [7], have been shown to be able to elevate Chl levels and promote plant growth, regardless of pathogen attack. Root inoculation with *B. amyloliquefaciens* Bs006 or *P. fluorescens* Ps006 promoted banana growth and stress tolerance [106]. A global transcriptome analysis revealed that hundreds of genes involved in stress response are affected by both PGPR strains at 48 hpi, but only *B. amyloliquefaciens* Bs006 affected the expression of genes involved in thylakoid and photosynthesis [106]. These results highlight that photosynthesis modification is one way, but not the only way, that PGPR offsets fitness costs.

## 4. Conclusions

**Figure 5:**
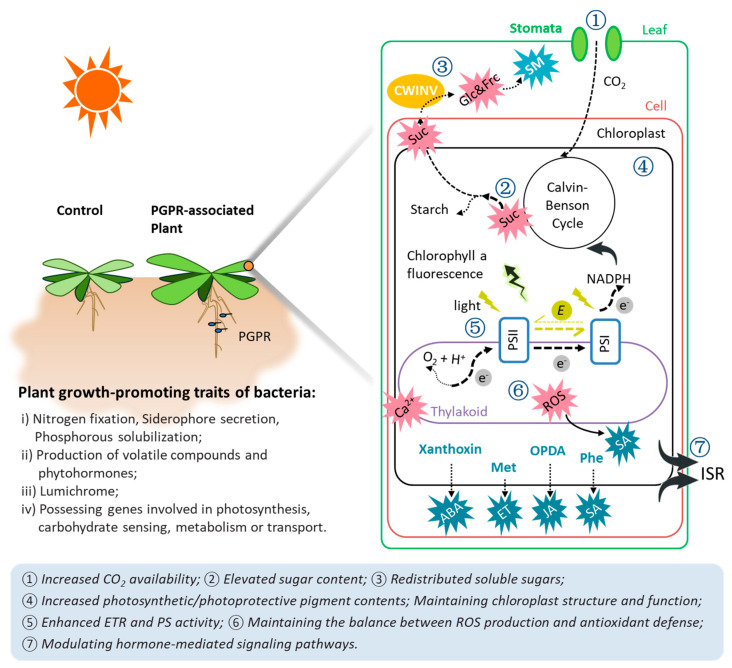
Simplified overview of PGPR-induced effects on photosynthesis. Plants associated with PGPR are always greener than controls due to PGPR-induced higher leaf pigment levels. In summary: (i) PGPR facilitates soil element absorption and utilization by plants through nitrogen fixation, siderophore secretion, or Phosphorous solubilization. Those essential elements are involved in the synthesis of pigments, thylakoids, and chloroplasts (photosynthetic apparatus). (ii) Moreover, photosynthetic or photoprotective pigment accumulations are also modulated by ABA, bacterial volatile compounds, and plant circadian rhythms. PGPR-induced accumulation on pigments and photosynthetic apparatus could lead to enhanced photosynthesis and prevent harm caused by stress to the site of photosynthesis. (iii) Secretion of lumichrome enhances root respiration and may increase CO_2_ availability for photosynthetic carbon assimilation. (iv) PGPR can be directly related to host plant carbon flux from photosynthesis; however, increased soluble sugar levels are usually detected in bacterized plants. Stimulated energy production by PGPR is required not only for plant growth but also for activation of plant defense responses. Full arrows depict interactions, broken arrows transport, and dotted arrows metabolic reactions. Abbreviations: ABA—abscisic acid; Ca^2+^—calcium; CWINV—cell wall invertase; E—photon; e^−^—electron; ET—ethylene; ETR—electron transport rate; Frc—fructose; Glc—glucose; ISR—induced systemic resistance; JA—jasmonic acid; NADPH—nicotinamide adenine dinucleotide phosphate; PGPR—plant-growth-promoting rhizobacteria; PSI—photosystem I; PSII—photosystem II; ROS—reactive oxygen species; SA—salicylic acid; SM—secondary metabolites; Suc—sucrose.

The critical role of PGPR in enhancing plant disease resistance is undeniable. The surge in consumer awareness and demand for pesticide-free food products presents a promising outlook for more commercially available PGPR strains. Screening and successful use of PGPR that can enhance photosynthesis could be a cost-saving strategy to improve crop yields under unpredictable and variable pathogen stress (Figure 5). It is relatively easy to evaluate the potential of PGPR to secrete plant hormones and other related compounds to promote plant growth by comparative and functional analyses of multiple PGPR genomes. In contrast, rhizobacteria’s effects on photosynthesis and plant stress responses are more like a symphony of internal and external factors of the bacterium and its host plants. The complexity of this interaction relies not only on the PGPR species used and the mode but also on the timing of PGPR application. Thus, it is important to investigate in more detail how PGPR modulates carbohydrate metabolism in connection with defense mechanisms. We reviewed photosynthetic alterations induced by PGPR with or without pathogen challenges and potentially important influencing factors. We highlight that PGPR-induced modulation of photosynthesis and carbohydrate metabolism has a significant role in triggering plant immune responses and developing ISR against pathogen invasion.

The potential benefits of PGPR-induced modifications in photosynthesis and carbohydrate distribution for agricultural practices are significant. Integrating PGPR treatments can enhance photosynthetic efficiency, increase disease resistance, and improve overall yield, providing eco-friendly alternatives to chemical pesticides. In regions facing challenges such as climate change and emerging plant diseases, PGPR-induced photosynthesis enhancement plays a crucial role in improving biotic resilience under environmental stress. Evaluating the economic viability of PGPR-treated crops, considering production costs and market demand, is vital for successful implementation. In conclusion, unraveling PGPR-induced alterations in carbohydrate metabolism supports sustainable agriculture and addresses global food security challenges.

## Data Availability

Not applicable.
